# Supplementary material for: Elevated Expression of HSP72 in the Prefrontal Cortex and Hippocampus of Rats Subjected to Chronic Mild Stress and Treated with Imipramine
Source: Int J Mol Sci. 2023 Dec 23;25(1):243. doi: 10.3390/ijms25010243 (PMC10779295; doi:10.3390/ijms25010243)
Supplement: Supplementary file 1 [file ijms-25-00243-s001.zip › Supplementary Tables S1-S3.pdf]

Elevated expression of HSP72 in the prefrontal cortex and hippocampus of rats subjected to chronic mild stress and treated with imipramine

Bielawski A.[1], Zelek-Molik A.[1], Rafa-Zabłocka K.[1], Kowalska M.[1], Gruca P.[2], Papp M.2 and Nalepa I.[1]

[1] Department of Brain Biochemistry, Maj Institute of Pharmacology, Polish Academy of Sciences, Kraków, Poland;

[2] Behavioral Pharmacology Laboratory, Maj Institute of Pharmacology, Polish Academy of Sciences, Kraków, Poland;

Supplementary Table S1. Lack of changes in expression of HSC70 mRNA in PFC and HIP of rats subjected to the CMS (stress) for 8 weeks and treated with IMI (10 mg/kg, ip) for 5 weeks determined by RTqPCR.

Treatment	**HSC70** mRNA level [% of sal/sham control ± SEM] [1]	
	**PFC**	**HIP**
sham/sal	100,00 ± 21,14	100,00 ± 7,69
stress/sal	88,95 ± 22,11	107,79 ± 3,66
sham/IMI	83,96 ± 14,87	91,40 ± 6,75
stress/IMI resp	96,63 ± 17,67	97,95 ± 4,17
stress/IMI nonresp	79,81 ± 5,98	107,40 ± 7,57
1 way ANOVA	*F(4,25) = 0.88, p > 0.05*	*F(4,25) = 1.23, p >0.05*

[1] Results are expressed as a percent of control (sham/sal) group ± standard error of mean (SEM); n = 6, stress/IMI resp – group of CMS rats that responded to IMI treatment (reversed anhedonia in sucrose test), stress/IMI nonresp – group of CMS rats nonresponding to IMI.

Supplementary Table S2. Lack of changes in expression of mRNAs of HSP90 family in the prefrontal cortex and HSP90A in the hippocampus of rats subjected to the CMS (stress) during 8 weeks and treated with IMI (10 mg/kg, ip) for 5 weeks determined by RTqPCR

Treatment	mRNAs level [% of sal/sham control ± SEM] [1]		
	**PFC**		**HIP**
	**HSP90A**	**HSP90B**	**HSP90A**
sham/sal	100,00± 7,39	100,00 ± 5,20	100,00 ± 1,80
stress/sal	100,80 ± 8,78	100,85 ± 5,86	104,98 ± 3,90
sham/IMI	109,89 ± 7,37	101,33 ± 4,24	98,07 ± 3,59
stress/IMI resp	101,45 ± 3,75	111,74 ± 6,95	102,90 ± 3,44
stress/IMI nonresp	97,86 ± 5,12	104,32 ± 6,17	100,62 ± 4,34
1 way ANOVA	*F(4,25) = 0.47, p > 0.05*	*F(4,25) = 1.29, p > 0.05*	*F(4,25) = 0.58, p > 0.05*

[1]Results are expressed as a percent of control (sham/sal) group ± standard error of mean (SEM); n = 6, stress/IMI resp – group of CMS rats that responded to IMI treatment (reversed anhedonia in sucrose test), stress/IMI nonresp – group of CMS rats nonresponding to IMI.

Supplementary Table S3. Lack of changes in mRNA expression of HSPA and HSPC families in Thal of rats subjected to the CMS (stress) for 8 weeks and treated with IMI (10 mg/kg, ip) for 5 weeks determined by RTqPCR.

Treatment	mRNAs level in **Thal** [% of sal/sham control ± SEM][1]			
	**HSP72**	**HSC70**	**HSP90A**	**HSP90B**
sham/sal	100,00 ± 7,50	100,00 ± 3,20	100,00 ± 11,30	100,00 ± 2,89
stress/sal	95,60 ± 8,43	109,00 ± 4,50	105,99 ± 5,38	99,01 ± 3,87
sham/IMI	99,80 ± 4,93	101,90 ± 4,70	108,00 ± 4,86	101,84 ± 4,12
stress/IMI resp	97,07 ± 9,94	112,00 ± 9,10	101,25 ± 7,51	97,88 ± 8,39
stress/IMI nonresp	115,74 ± 8,34	99,00 ± 5,00	100,72 ± 5,32	99,01 ± 7,58
1 way ANOVA	$F(4,25) = 1.05$, $p > 0.05$	$F(4,25) = 1.04$, $p > 0.05$	$F(4,25) = 0.23$, $p > 0.05$	$F(4,25) = 0.07$, $p > 0.05$

[1]Results are expressed as a percent of control (sham/sal) group ± standard error of mean (SEM); n = 6, stress/IMI resp – group of CMS rats that responded to IMI treatment (reversed anhedonia in sucrose test), stress/IMI nonresp – group of CMS rats nonresponding to IMI.
