# Supplementary material for: Elevated Expression of HSP72 in the Prefrontal Cortex and Hippocampus of Rats Subjected to Chronic Mild Stress and Treated with Imipramine
Source: Int J Mol Sci. 2023 Dec 23;25(1):243. doi: 10.3390/ijms25010243 (PMC10779295; doi:10.3390/ijms25010243)

Elevated expression of HSP72 in the prefrontal cortex and hippocampus of rats subjected to chronic mild stress and treated with imipramine

Bielawski A.[1], Zelek-Molik A.[1], Rafa-Zabłocka K.[1], Kowalska M.[1], Gruca P.[2], Papp M.2 and Nalepa I.[1]

[1] Department of Brain Biochemistry, Maj Institute of Pharmacology, Polish Academy of Sciences, Kraków, Poland;

[2] Behavioral Pharmacology Laboratory, Maj Institute of Pharmacology, Polish Academy of Sciences, Kraków, Poland;

Western blot images corresponding to Figures 2B., 3B., 4B.

After protein transfer and membrane staining with PonceauS, membranes were cut horizontally on ~62 kDa, then Hsp72 and Hsp90B were assessed on the upper piece while loading control (beta-actin or alpha-tubulin) on the bottom part. The molecular weight of studied proteins was checked in the initial experiment (performed for validation/optimization of the amount of Ab/protein taken for the experiment) using molecular markers (BioRad). Only good-quality bands were taken for analysis.

Fig. 2B. Hsp72 protein

Prefrontal cortex

Gel #5

band# from left	1	2	3	4	5	6	7	8	9	10
group	sham/sal	stress/sal	sham/IMI	stress/IMI resp	stress/IMI non-resp	sham/sal	stress/sal	sham/IMI	stress/IMI resp	stress/IMI non-resp
sample#	11	21	31	41	51	12	22	32	42	52
band# from left	11	12	13	14	15	16	17	18	19	20
group	sham/sal	stress/sal	sham/IMI	stress/IMI resp	stress/IMI non-resp	sham/sal	stress/sal	sham/IMI	stress/IMI resp	stress/IMI non-resp
sample#	13	23	33	43	53	14	24	34	44	54
band# from left	21	22	23	24						
group	sham/sal	stress/sal	sham/IMI	stress/IMI resp						
sample#	15	25	35	45						

Hsp72 (Ms) ~70 kDa band indicated by the blue arrow

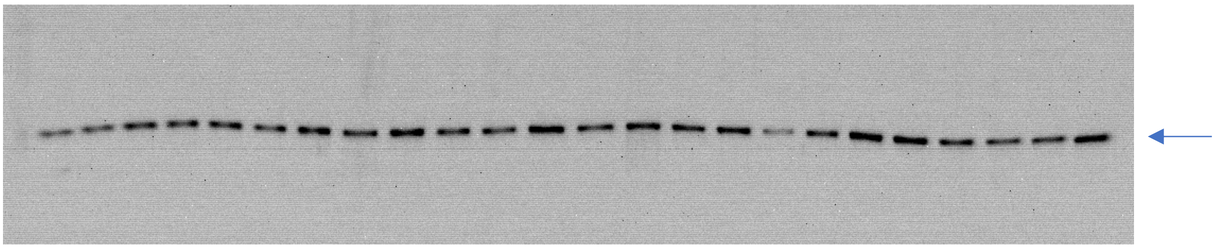

alpha-tubulin (Ms) ~55kDa band indicated by red arrow

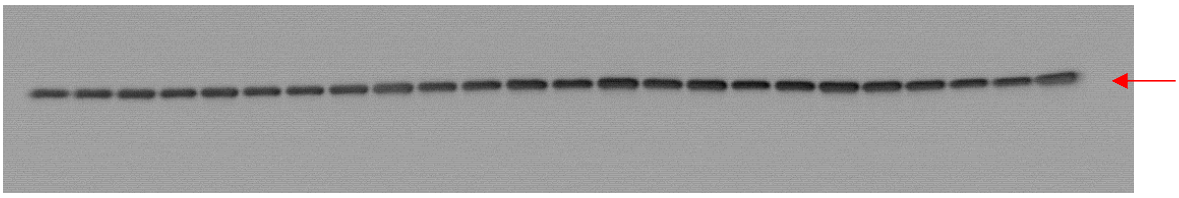

Gel I#6

band# from left	1	2	3	4	5	6	7	8	9	10
group	sham/sal	stress/sal	sham/IMI	stress/IMI resp	stress/IMI non-resp	sham/sal	stress/sal	sham/IMI	stress/IMI resp	stress/IMI non-resp
sample#	16	26	36	46	56	17	27	37	47	57
band# from left	11	12	13	14	15					
group	sham/sal	stress/sal	sham/IMI	stress/IMI resp	stress/IMI non-resp					
sample#	18	28	38	48	58					

Hsp72 (Ms) ~70 kDa band indicated by the blue arrow

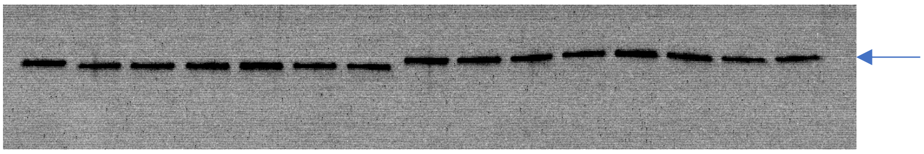

alpha-tubulin (Ms) ~55kDa band indicated by red arrow

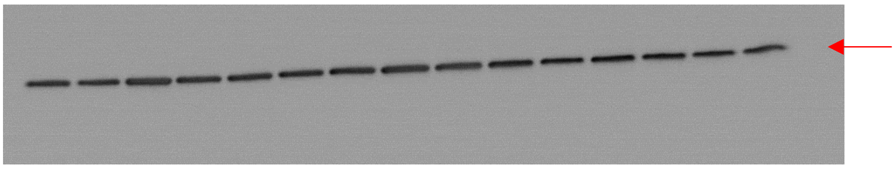

Fig. 3B. Hsp72 protein

Hippocampus

Gel #3

band# from left	1	2	3	4	5	6	7	8	9	10
group	sham/sal	sham/sal	stress/sal	stress/sal	sham/IMI	sham/IMI	stress/IMI resp	stress/IMI resp	stress/IMI non-resp	stress/IMI non-resp
sample#	11	12	21	22	31	32	41	42	51	52

Hsp72 (Ms) ~70 kDa band indicated by the blue arrow

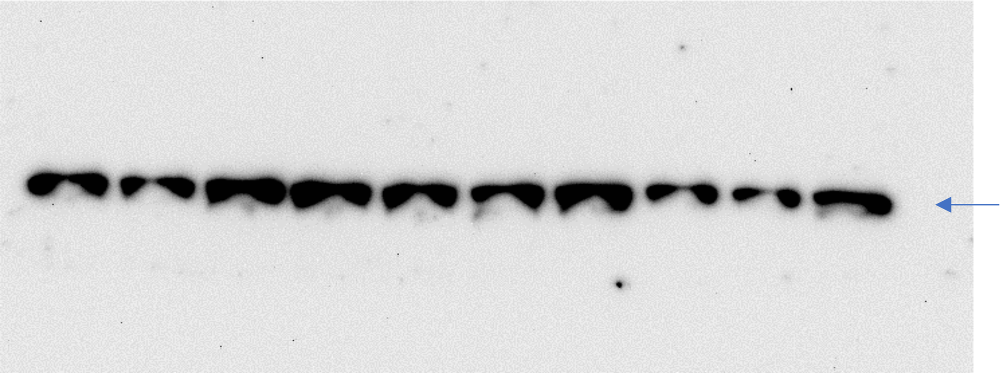

alpha-tubulin (Ms) ~55kDa band indicated by red arrow

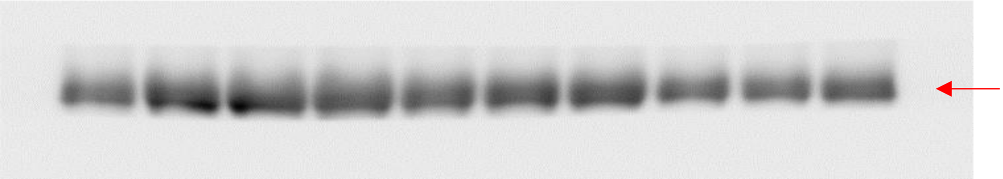

Gel #4

band# from left	1	2	3	4	5	6	7	8	9	10
group	sham/sal	sham/sal	stress/sal	stress/sal	sham/IMI	sham/IMI	stress/IMI resp	stress/IMI resp	stress/IMI non-resp	stress/IMI non-resp
sample#	13	14	23	24	33	34	43	44	53	54

Hsp72 (Ms) ~70 kDa band indicated by the blue arrow

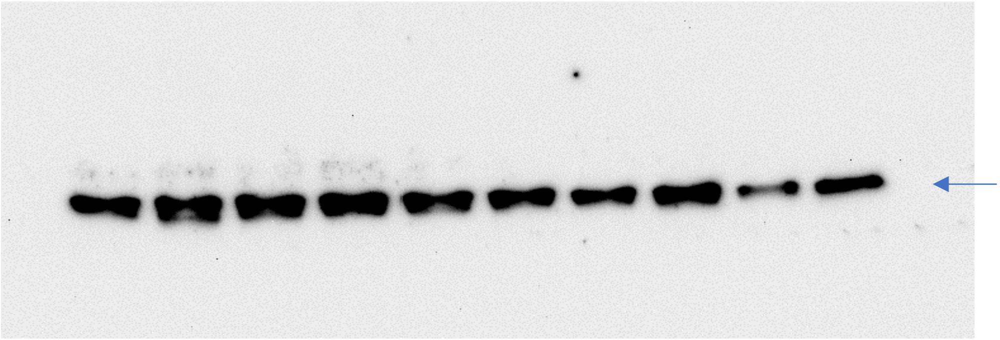

alpha-tubulin (Ms) ~55kDa band indicated by red arrow

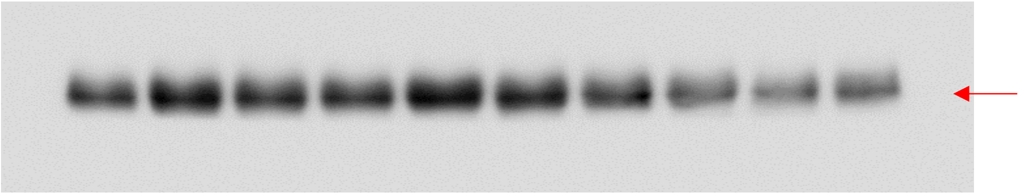

Gel #5

band# from left	1	2	3	4	5	6	7	8	9	10
group	sham/sal	sham/sal	stress/sal	stress/sal	sham/IMI	sham/IMI	stress/IMI resp	stress/IMI resp	stress/IMI non-resp	stress/IMI non-resp
sample#	15	16	25	26	35	36	45	46	55	56

Hsp72 (Ms) ~70 kDa band indicated by the blue arrow

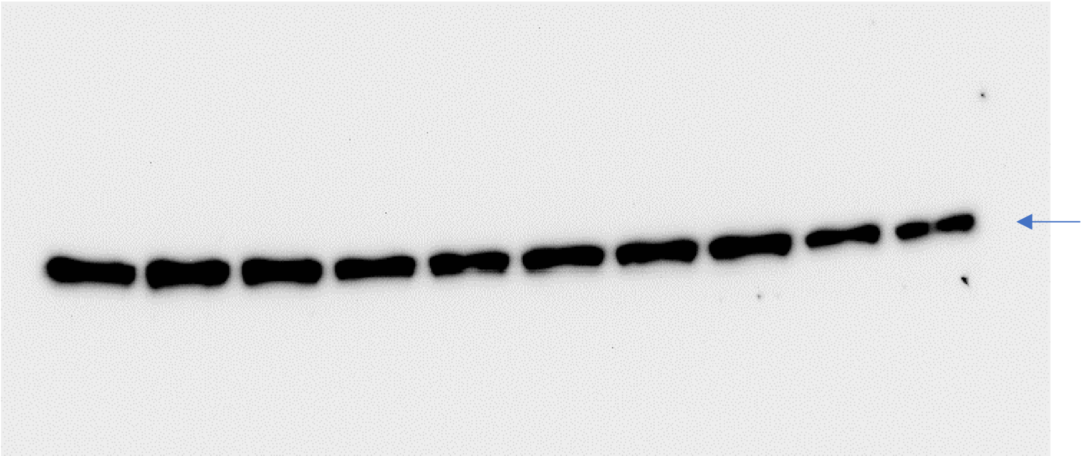

alpha-tubulin (Ms) ~55kDa band indicated by red arrow

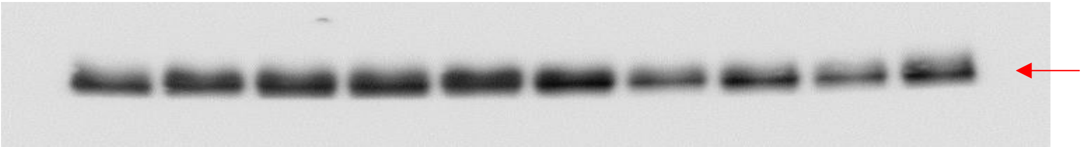

Fig. 4B. Hsp90B protein

Hippocampus

Gel #1

band# from left	1	2	3	4	5	6	7	8	9	10
group	sham/sal	stress/sal	sham/IMI	stress/IMI resp	stress/IMI non-resp	sham/sal	stress/sal	sham/IMI	stress/IMI resp	stress/IMI non-resp
sample#	11	21	31	41	51	12	22	32	42	52
band# from left	11	12	13	14	15	16	17	18	19	20
group	sham/sal	stress/sal	sham/IMI	stress/IMI resp	stress/IMI non-resp	sham/sal	stress/sal	sham/IMI	stress/IMI resp	stress/IMI non-resp
sample#	13	23	33	43	53	14	24	34	44	54
band# from left	21	22	23	24	25					
group	sham/sal	stress/sal	sham/IMI	stress/IMI resp	stress/IMI non-resp					
sample#	15	25	35	45	55					

Hsp90B (Ms) ~85kDa band indicated by blue arrow

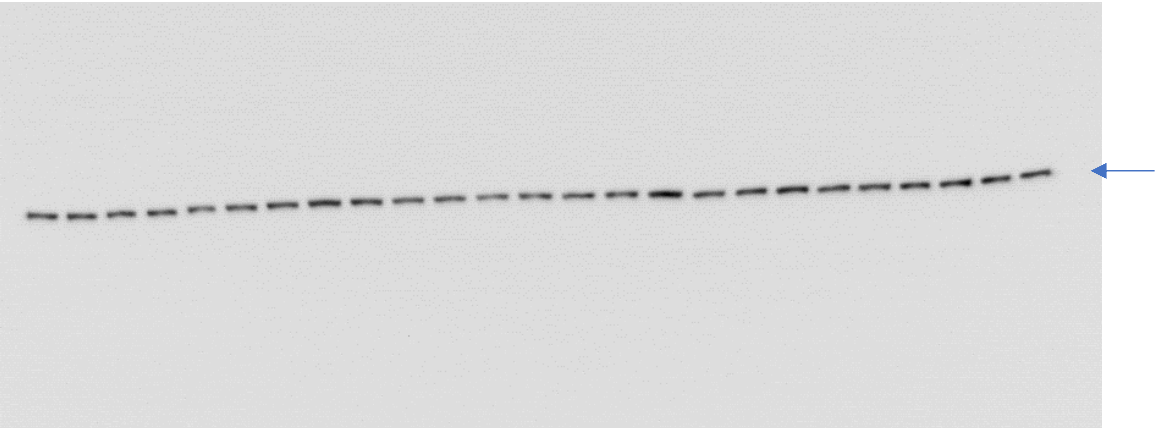

beta-actin (Ms) ~43kDa band indicated by red arrow

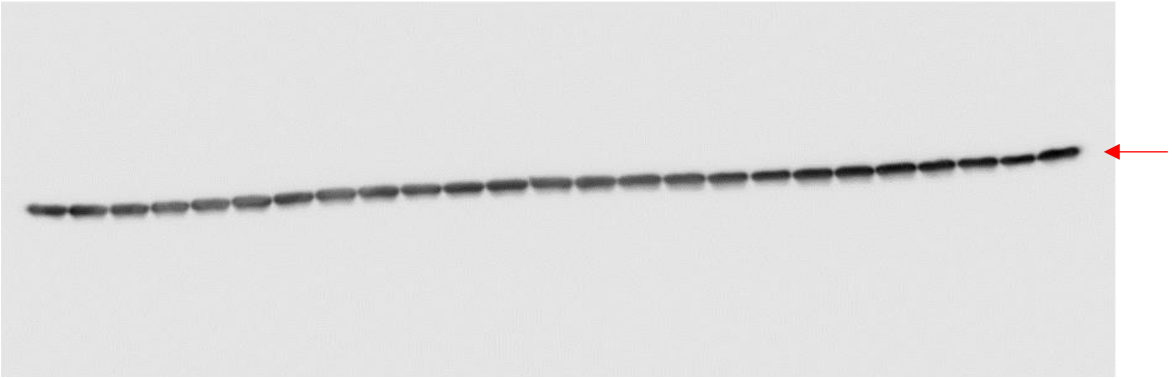

Gel #2

band# from left	1	2	3	4	5	6	7	8	9	10
group	sham/sal	stress/sal	sham/IMI	stress/IMI resp	stress/IMI non-resp	sham/sal	stress/sal	sham/IMI	stress/IMI resp	stress/IMI non-resp
sample#	16	26	36	46	56	17	27	37	47	57

band# from left	11	12	13	14	15
group	sham/sal	stress/sal	sham/IMI	stress/IMI resp	stress/IMI non-resp
sample#	18	28	38	48	58

Hsp90B (Ms) ~85kDa band indicated by blue arrow

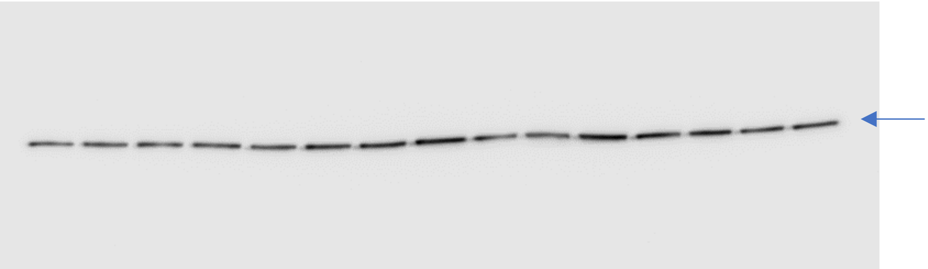

beta-actin (Ms) ~43kDa band indicated by red arrow

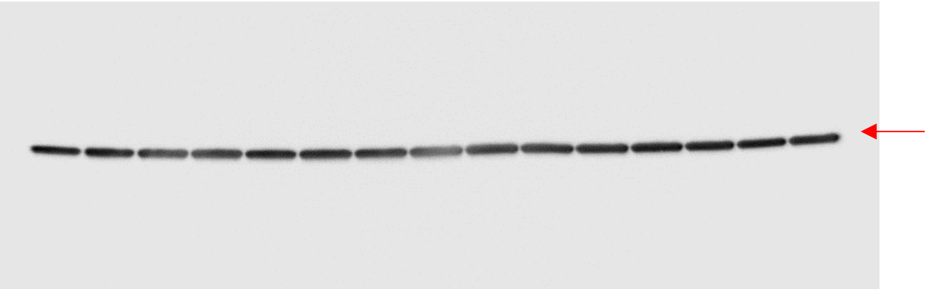

Supplement: Supplementary file 1 [file ijms-25-00243-s001.zip › Supplementary_Figure_S1.pdf]
