# Supplementary figures and images for: Elevated Expression of HSP72 in the Prefrontal Cortex and Hippocampus of Rats Subjected to Chronic Mild Stress and Treated with Imipramine
Source: Int J Mol Sci. 2023 Dec 23;25(1):243. doi: 10.3390/ijms25010243 (PMC10779295; doi:10.3390/ijms25010243)

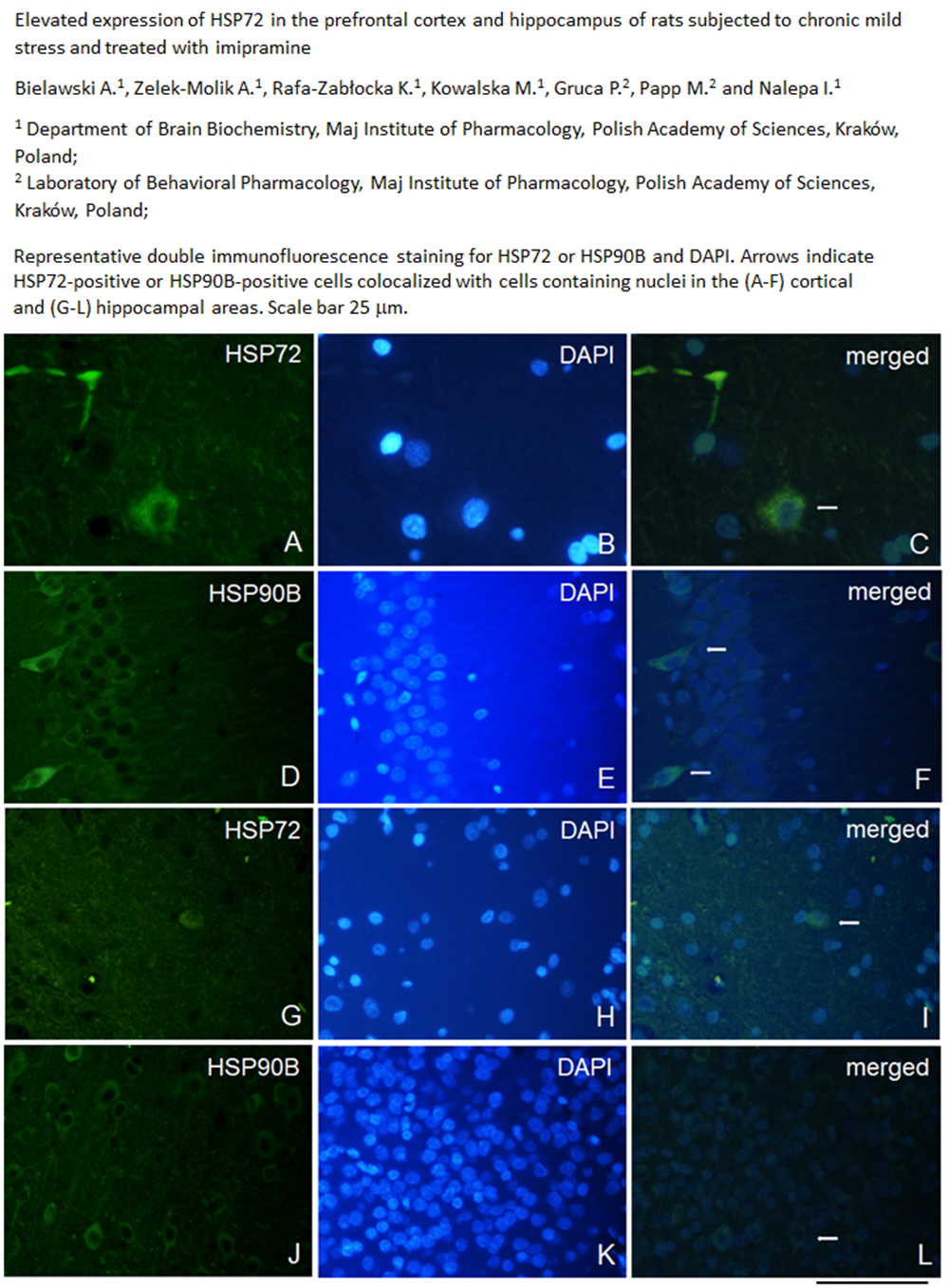

Supplement: Supplementary file 1 [file ijms-25-00243-s001.zip › Supplementary_Figure_S2.jpg]

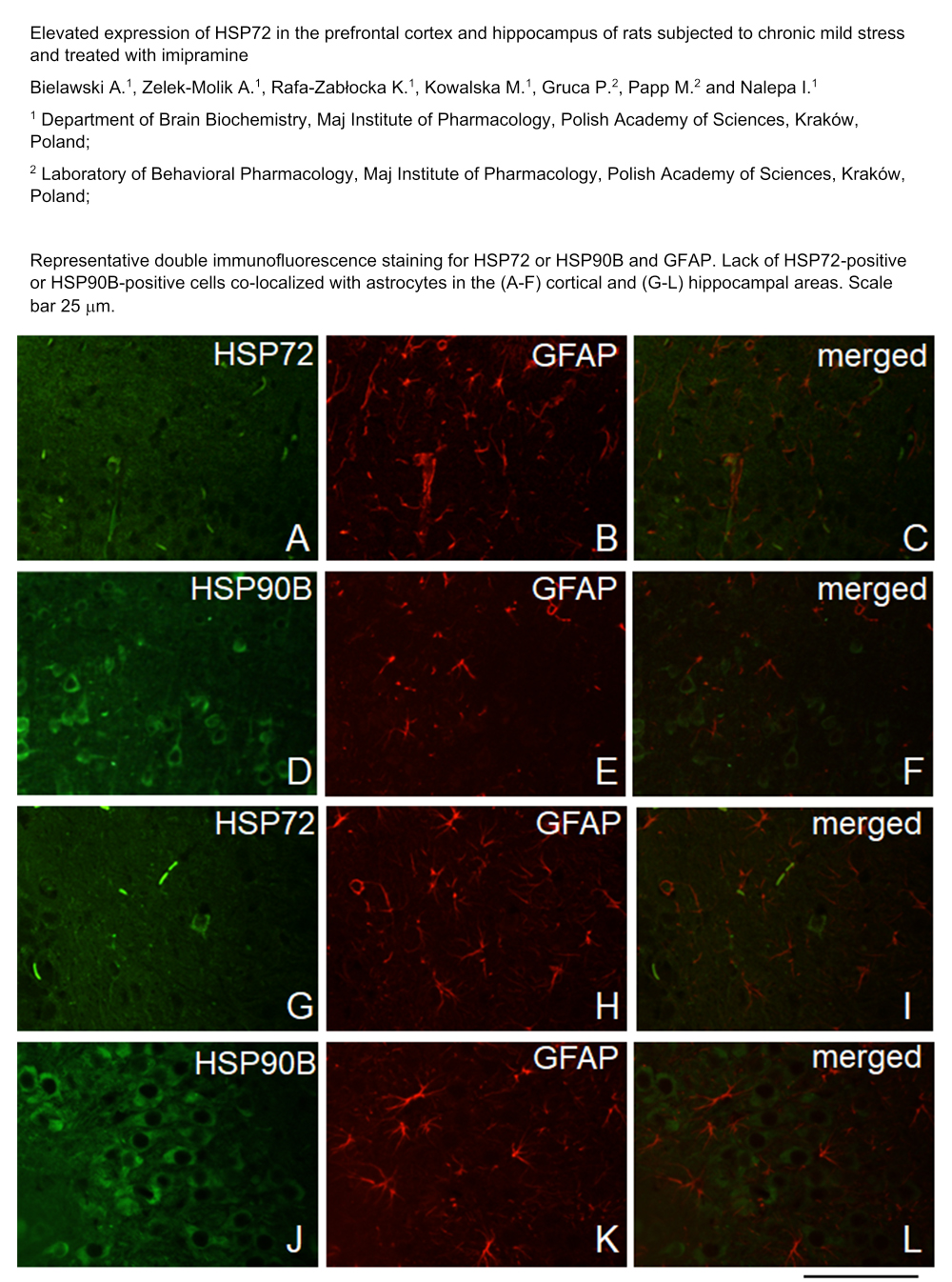

Supplement: Supplementary file 1 [file ijms-25-00243-s001.zip › Supplementary_Figure_S3.jpg]
